# Supplementary material for: Electric-field control of skyrmions in multiferroic heterostructure via magnetoelectric coupling
Source: Nat Commun. 2021 Jan 12;12:322. doi: 10.1038/s41467-020-20528-y (PMC7803786; doi:10.1038/s41467-020-20528-y)
Supplement: Supplementary file 1 — Supplementary Information [file 41467_2020_20528_MOESM1_ESM.pdf]

Electric-field control of skyrmions in multiferroic heterostructure via magnetoelectric coupling

You Ba[1,2†], Shihao Zhuang[3†], Yike Zhang[1,2†], Yutong Wang[1,2], Yang Gao[4], Hengan Zhou[1,2], Mingfeng Chen[5], Weideng Sun[1,2], Quan Liu[1,2], Guozhi Chai[4], Jing Ma[5], Ying Zhang[6], Huanfang Tian[6], Haifeng Du[7], Wanjun Jiang[1,2], Cewen Nan[5], Jia-Mian Hu[3★] and Yonggang Zhao[1,2★]

[1]Department of Physics and State Key Laboratory of Low-Dimensional Quantum Physics, Tsinghua University, Beijing 100084, China

[2]Frontier Science Center for Quantum Information, Tsinghua University, Beijing 100084, China

[3]Department of Materials Science and Engineering, University of Wisconsin-Madison, WI 53706, Madison

[4]Key Laboratory for Magnetism and Magnetic Materials of the Ministry of Education, Lanzhou University, Lanzhou 730000, China

[5]School of Materials Science and Engineering and State Key Lab of New Ceramics and Fine Processing, Tsinghua University, Beijing 100084, China

[6]Beijing National Laboratory for Condensed Matter Physics, Institute of Physics, Chinese Academy of Sciences, Beijing 100190, China

[7]Anhui Province Key Laboratory of Condensed Matter Physics at Extreme Conditions, High Magnetic Field Laboratory of Chinese Academy of Sciences, China

[†]These authors contributed equally to this work.

[★]Corresponding author. E-mail: ygzhao@tsinghua.edu.cn; jhu238@wisc.edu

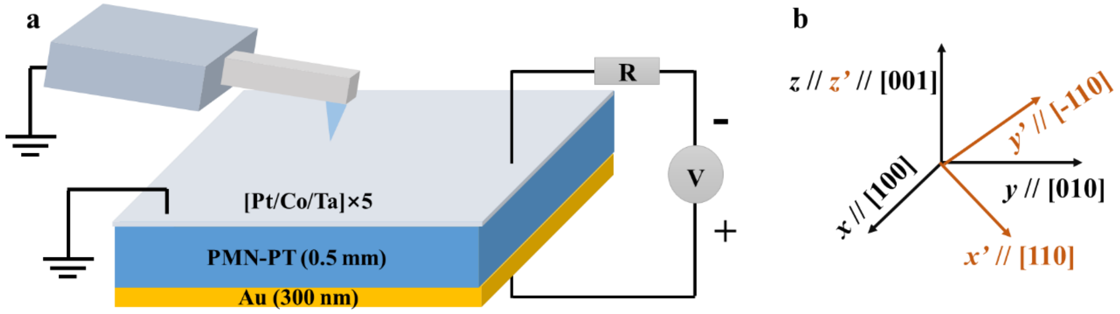

Supplementary Fig. 1 | Schematic of the sample configuration. a, Schematic of the sample stack structure and set up for MFM measurement with *in situ* electric fields. **b,** Coordinate systems defined for experiment (black) and simulations (brown).

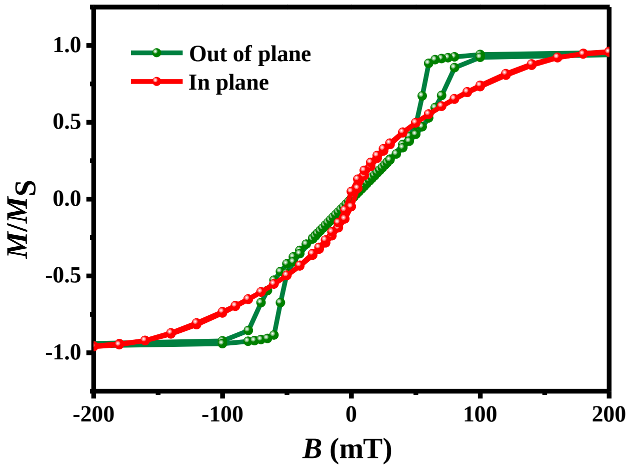

Supplementary Fig. 2 | Magnetic hysteresis loops. Normalized out-of-plane (olive) and in-plane (red) magnetic hysteresis loops of the sample.

Several magnetic parameters can be obtained from the hysteresis loop: saturation magnetization, $M_S = 9.48\times10^5$ A/m, the effective magnetic anisotropy field, $\mu_0 H_K = 180$ mT (μ_0 is the vacuum permeability), defined as the crossing point of the out-of-plane and in-plane magnetic hysteresis loops. Therefore, the uniaxial magnetic anisotropy constant is $K_U = \frac{1}{2}\mu_0 \mathbf{H}_K \cdot \mathbf{M}_S + \frac{1}{2}\mu_0 \mathbf{M}_S{}^2 = 6.5\times10^5\ \mathrm{J/m^3}$.

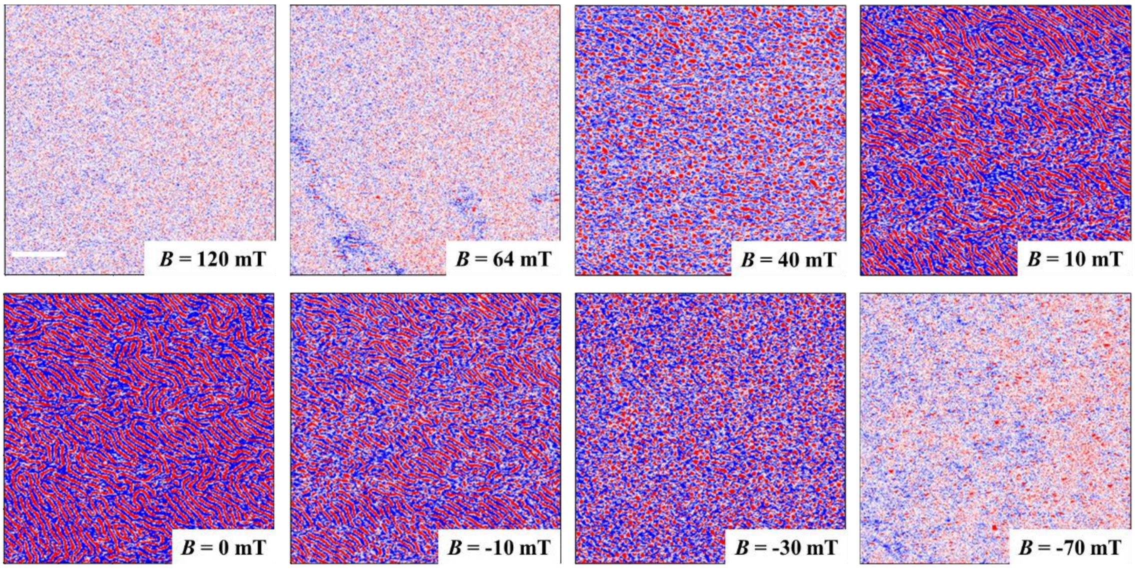

Supplementary Fig. 3 | MFM observation of evolutions of the magnetic domain by varying magnetic field from the positive saturation field to the negative one along the perpendicular direction. The scale bar is 2 μm.

Initially, the ferromagnetic saturation state was observed in the MFM image without contrast at 120 mT, larger than the out-of-plane saturation field (100 mT). Then, skyrmions appear gradually when reducing magnetic field to 64 mT, and an entire skyrmion state is reached at 40 mT. After that, the skyrmions begin to merge together and transform to stripe domains and labyrinth domains at 0 mT. Continue to increase magnetic field in the opposite direction, skyrmions reappear from the labyrinth domains, and reach the maximum skyrmion density and then change to magnetization saturation state, similar to the decreasing magnetic field process.

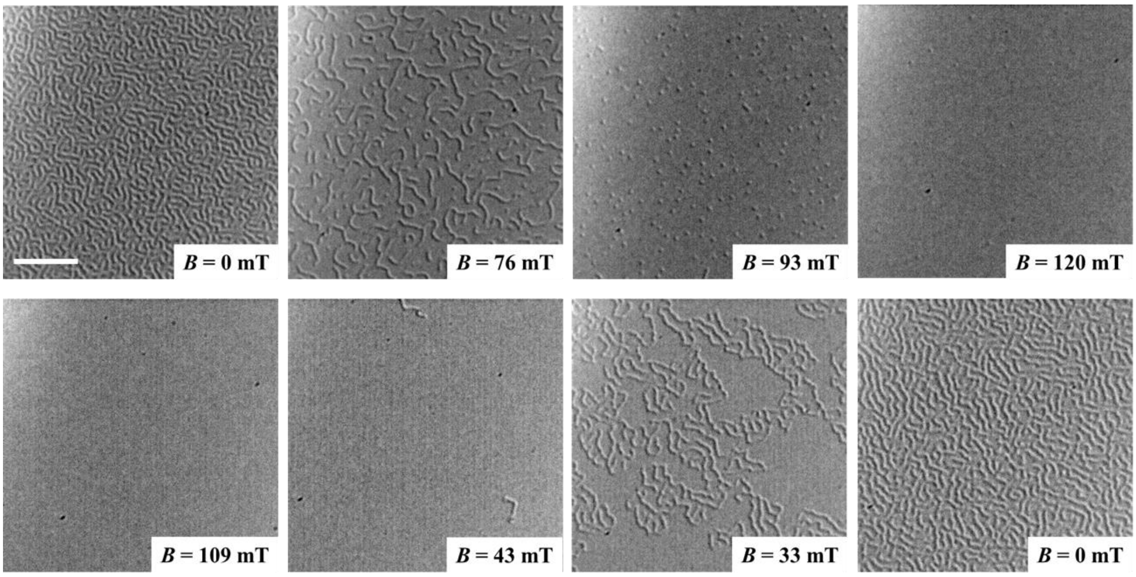

Supplementary Fig. 4 | L-TEM observation of evolutions of the magnetic domain by increasing and decreasing the perpendicular magnetic fields. The scale bar is 2 μm.

When increasing magnetic field, the maximum skyrmion density was obtained at 93 mT, and no skyrmion appears when decreasing magnetic field, which is different from the skyrmion evolutions characterized by MFM. The difference is likely attributed to the different magnetic properties in different substrates used in the two characterizations.

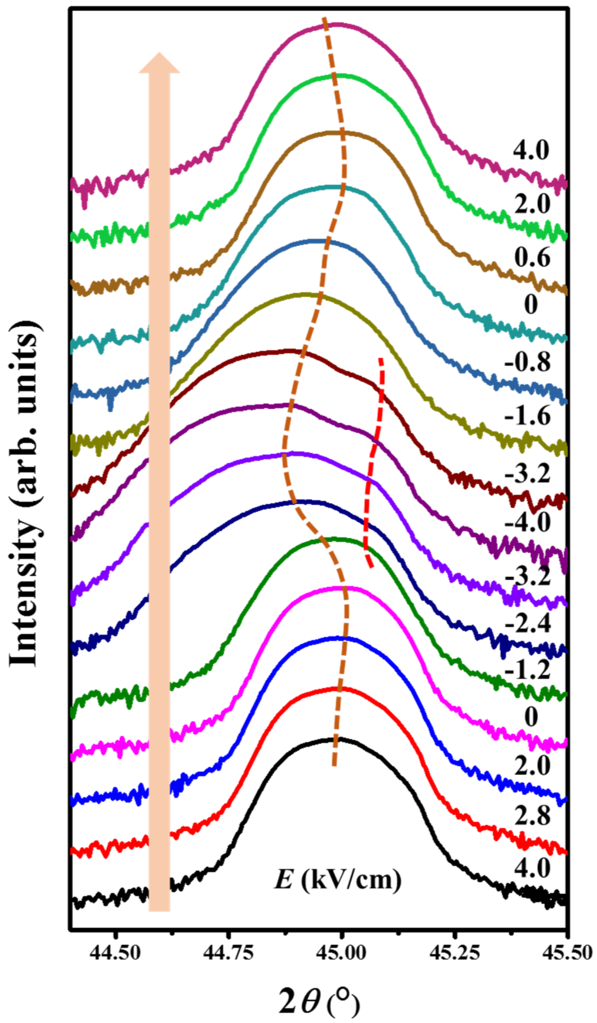

Supplementary Fig. 5 | XRD data of the (002) peak of PMN-PT(001) substrate under different electric fields.

The (002) diffraction peak of PMN-PT(001) FE single crystal moves to the left side slightly under positive electric fields, and has an obvious shift after applying negative electric fields. Meanwhile, it shows prominent broadening and splitting after -1.2 kV/cm, which hints a ferroelectric phase transformation in our sample[1]. Generally, PMN-PT(001) single crystal near the morphotropic phase boundary (MPB) has rich ferroelectric phases, and is more likely to transform between the different ferroelectric phases under electric field[1]. Such characteristic is the main cause for the large piezoelectric effect[2], and may also explain the exotic strain behavior in our sample.

Nevertheless, the discussion of the detailed ferroelectric phase transformation is beyond our scope, thus in the following, we focus on the influence of the strain on the skyrmions, without further exploring the strain origin.

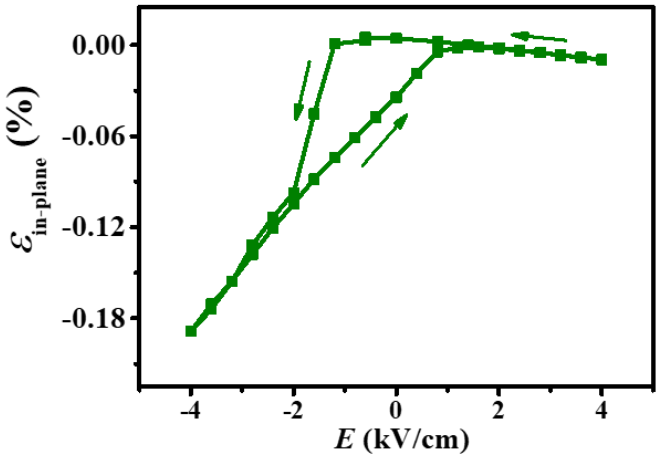

Supplementary Fig. 6 | In-plane isotropic biaxial strain. Electric field dependence of the in-plane isotropic compressive strain curve is deduced by $\varepsilon_{\text{in-plane}} = \varepsilon_{[110]} = \varepsilon_{[-110]} = \nu_{\text{PMN-PT}} \cdot \varepsilon_{[001]}^{\text{PMN-PT}}$ with $\nu_{\text{PMN-PT}} = -0.5$ (ref. 3), where $\varepsilon_{[001]}^{\text{PMN-PT}}$ is the out-of-plane strain of PMN-PT substrate shown in Fig. 2a.

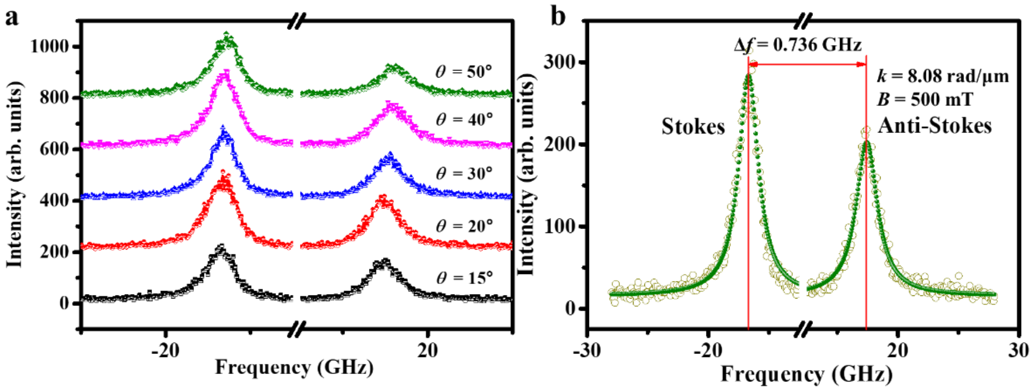

Supplementary Fig. 7 | BLS spectra. a, Wave-vector dependence of BLS spectra of

our sample. In-plane wave-vector k is determined by $k = \frac{4\pi}{\lambda}\sin\theta$, where λ is the wave length of 532 nm, θ is the angle between incident light and z axis. **b,** An example of BLS spectrum with $k = 8.08\,\text{rad}/\mu\text{m}\,(\theta = 20°)$. The open dots are experimental data, and the solid dots represent Lorentzian fitting. The frequency shift between Stokes and Anti-Stokes is marked by the vertical lines, and $\Delta f = 0.736 \pm 0.014\,\text{GHz}$, with the standard error obtained from the Lorentzian fitting.

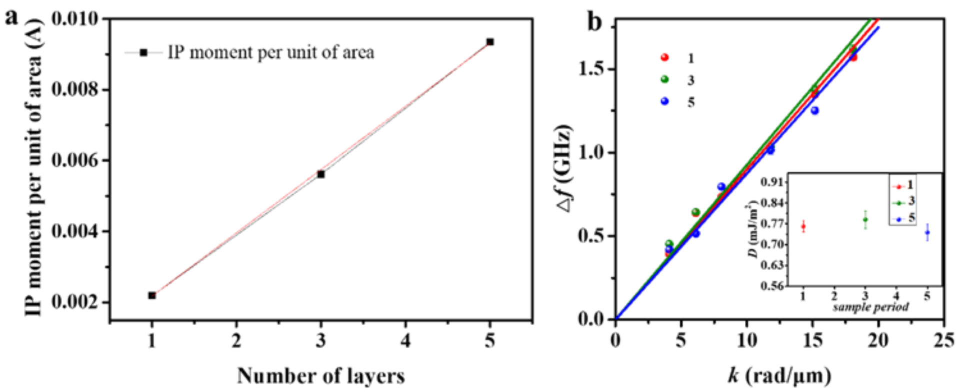

Supplementary Fig. 8 | In-plane saturation magnetic moments and interfacial DMI values of Si/Ta(4.7)/[Pt(4)/Co(1.6)/Ta(1.9)]n (n is 1, 3 and 5). a, The in-plane saturation magnetic moments are proportional to the number of period. As a result, it can be determined that the magnetic properties of each layer of our sample are equivalent. **b,** Wave-vector dependence of Δf in Si/Ta(4.7)/[Pt(4)/Co(1.6)/Ta(1.9)]n (n is 1, 3 and 5) with the interfacial DMI values in the inset with the error bar obtained from the standard error of Lorentzian fitting. Within the error range, the interfacial DMI values of the three samples are equivalent. The non-reciprocal effect due to the dipolar interaction is indeed pronounced in multilayers, however, it can be ignored for our work because the conditions for it to be effective are not satisfied. The conditions are the

magnetic layers are antiferromagnetically coupled, or ferromagnetically coupled with the magnetic properties (such as M_s) of the magnetic layers different. These conditions are required for the dipolar interactions in the multilayer film to give rise to the non-reciprocal effects (this theory proposed by Grungberg[11]). For our work, each magnetic layer has the same material and thickness, and we applied the in-plane saturation magnetic field of 5000 Oe during the measurement of BLS, so that the magnetic moments of the magnetic layers are parallel. In order to check whether the magnetic properties of each layer are equivalent, we grew Si/Ta(4.7)/$[Pt(4)/Co(1.6)/Ta(1.9)]_n$ multilayers with different periods (n is 1, 3 and 5, respectivley) by magnetic sputtering. It is found that their in-plane saturation magnetic moments are proportional to the number of period as shown in Fig. R9. As a result, it can be determined that the magnetic properties of each layer of our sample are equivalent. So it can be concluded that conditions for the dipolar interaction-induced non-reciprocal effect to be effective are not satisfied for our sample. For these reasons, the non-reciprocal effects due to dipolar interactions in our sample are negligible. Moreover, even if there is a weak non-reciprocal effect due to the dipolar interactions, considering that the sample's non-magnetic layer thickness is 5.9 nm, the non-reciprocal effects due to dipolar interactions decreases exponentially with the increase of the non-magnetic layer thickness[8], so these non-reciprocal effects can be ignored in our work.

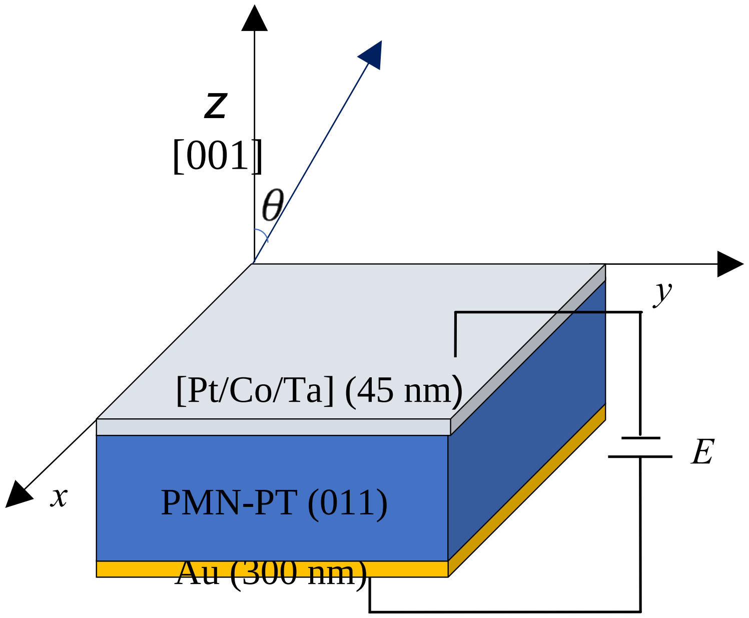

Supplementary Fig. 9 | Schematic of experimental configuration for angle-dependent FMR measurements.

We performed angle-dependent FMR measurements under different electric fields. As shown in Supplementary Fig. 9, θ is the angle between the applied magnetic field H and the out-of-plane (z) direction. For each θ, a resonance field $H_r(\theta)$ can be determined from the FMR spectrum. The magnetic anisotropy (denoted as K_{eff}) can be determined by fitting $H_r(\theta)$ with the Kittel formula for FMR.

Kittel formula for FMR can be written as following:

$$f = \frac{\gamma}{2\pi}\sqrt{A_1}\cdot\sqrt{A_2}$$

$$A_1 = H_r\cos(\theta-\theta_M) + H_1\cos^2\theta_M - H_2\cos^4\theta_M$$

$$A_2 = H_r\cos(\theta-\theta_M) + H_1\cos 2\theta_M + H_2\left(3\cos^2\theta_M\sin^2\theta_M - \cos^4\theta_M\right)$$

where $H_1 = 2K_{eff}/M_S + 4K_2/M_S$ and $H_2 = 4K_2/M_S$, γ is the gyromagnetic ratio given as $\gamma = g\mu_B/\hbar$, where g , μ_B and $\hbar$ are Lande's g factor , the Bohr magneton, and Planck's constant, respectively. θ_M is the angle between the

magnetization and the out-of-plane (z) direction. The saturation magnetizations (M_S) for different samples can be deduced from the magnetic hysteresis loops measured by MPMS. The three unknown quantities K_{eff} , K_2 and g can be achieved by fitting H_r versus θ using the Kittel formula.

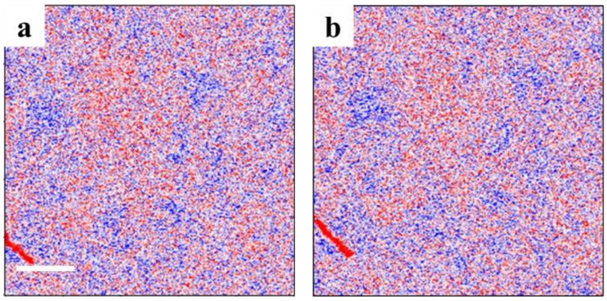

Supplementary Fig. 10 | Influence of the magnetic tip on sample. a, The first and **b,** second MFM scanning images with a scale bar of 1 μm. No skyrmions or other domain structures appear after the magnetic tip scanning, which indicates the influence of the magnetic tip can be ignored in this case. Moreover, the skyrmions creation is induced by the electric field as discussed in the Main Text.

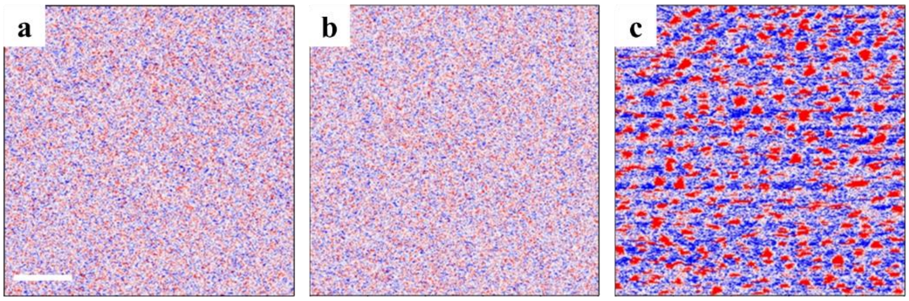

Supplementary Fig. 11 | Influence of the polarity of electric field. MFM images at E = -0 kV/cm (**a**), +4 kV/cm (**b**), -4 kV/cm (**c**), with a scale bar of 1 μm.

Initially, the sample has FM single domain and is in negatively polarized remnant

state (E = -0 kV/cm) with B_{bias} = 60 mT, shown in Supplementary Fig. 6a. When polarized by +4 kV/cm, there is no obvious change in the MFM image. However, the skyrmions are created at -4 kV/cm, as shown in Supplementary Fig. 11c. The skyrmions can only be created from the ferromagnetic saturation state when polarized by -4 kV/cm, consistent with the limited change under positive electric field in Fig. 3.

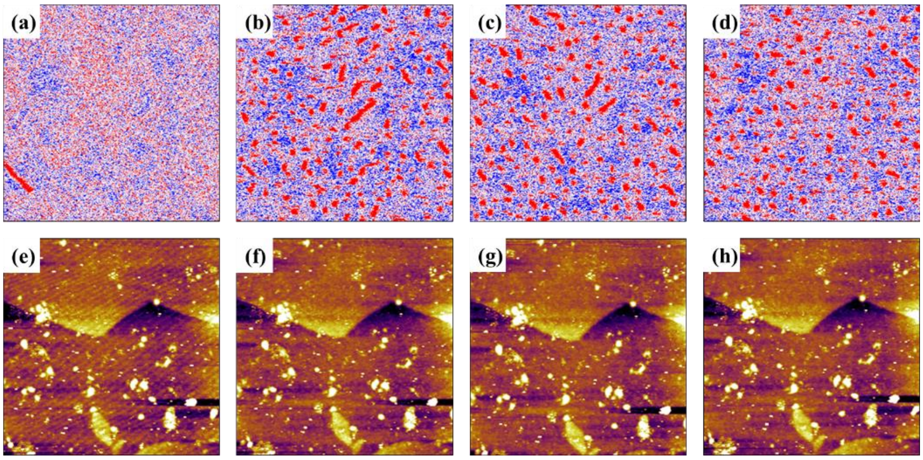

Supplementary Fig. 12 | MFM images at E = +0 kV/cm (a), -4 kV/cm (b), -0 kV/cm (c), +4 kV/cm (d) with B_{bias} = 60 mT and the corresponding topography images (e)-(h).

The MFM images were taken in the tapping/lift mode, i.e. the topography and magnetic images were obtained at the same time. The topography of the sample is obtained using the tapping mode for the first scan, and the magnetic image is obtained using the lift mode for the second scan. The magnetic images in Fig. 3 a-d and their corresponding topography images are shown in Supplementary Fig. 12. The similar topography images indicate that Fig. 3 a-d show the same location on the sample.

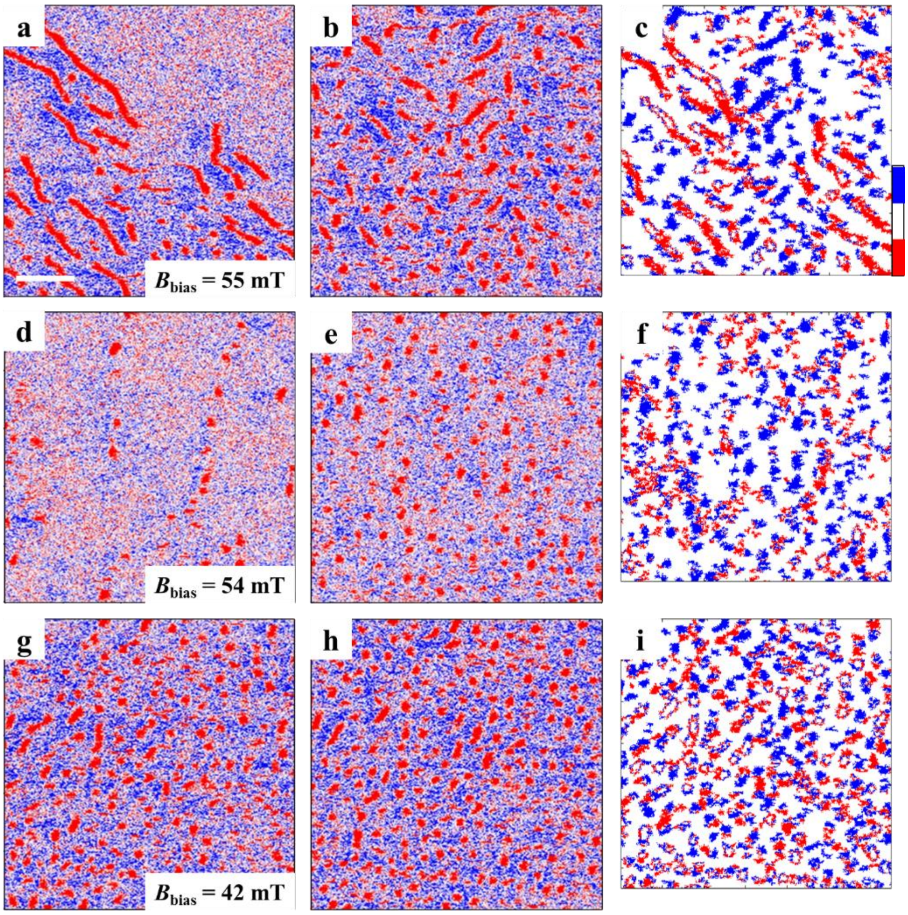

Supplementary Fig. 13 | Skyrmion manipulations under different bias magnetic fields. Three areas of the MFM images under different bias magnetic fields: **a, b,** B_{bias} = 55 mT; **d, e,** B_{bias} = 54 mT; **g, h,** B_{bias} = 42 mT. The first row for E = +0 kV/cm and the second row for E = -4 kV/cm. **c, f, i,** Point by point map difference between images at E = +0 kV/cm and E = -4 kV/cm, in which the blue contrast shows the appeared domain structure and the red contrast shows the disappeared one after polarized by -4 kV/cm. The scale bar is 1 μm.

At B_{bias} = 55 mT, the initial state shows a mixture of skyrmions and stripe domains in Supplementary Fig. 14a. When polarized by -4 kV/cm, more skyrmions are created from the saturation ferromagnetic background and the stripe domains also transform to skyrmions, which can also be indicated by the blue contrast and red stripe contrast in

map difference Supplementary Fig. 13c.

At B_{bias} = 54 mT, a few skyrmions are shown in Supplementary Fig. 13d and more skyrmions can be created by -4 kV/cm. The map difference, Supplementary Fig. 13f, shows more blue contrast than red ones.

At B_{bias} = 42 mT, the initial state shows skyrmions state with a large density, and little new skyrmions are created by -4 kV/cm. The blue and red contrasts in map difference, Supplementary Fig. 13i, are mainly due to the misalignment of the morphology.

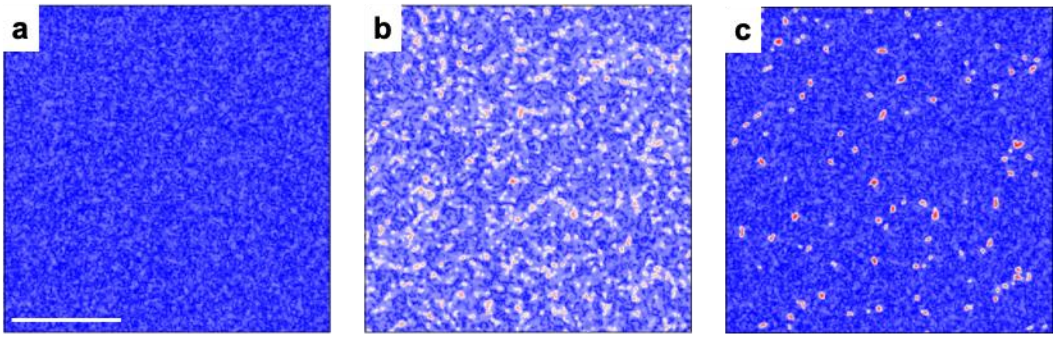

Supplementary Figure 14 | Retainment of skyrmions assuming strain is zero and D remains unchanged. a, Magnetization distribution at E = +0 kV/cm relaxed from an initially uniform [001] distribution under zero strains and D = 0.772 mJ/m^2. **b**, Multiple isolated skyrmions appeared after applying strain $\varepsilon_{[1-10]} = \varepsilon_{[110]} = -0.189\%$ and D = 0.585 mJ/m^2 at E = -4 kV/cm. **c**, Retainment of skyrmions at zero strain with D = 0.585 mJ/m^2 unchanged. The scale bar is 1 μm.

In the Main Text, the skyrmions were created by in-plane biaxial compressive strain at E = -4 kV/cm (Fig. 3f), and most of the skyrmions can be retained after removing electric field (E = -0 kV/cm) (Fig. 3g). Although there was remanent strain

of $\varepsilon_{[1-10]} = \varepsilon_{[110]} = -0.034\%$ and an enhancement of D from 0.585 mJ/m^2 to 0.685 mJ/m^2 at E = -0 kV/cm, neither this remanent strain nor the enhanced D is a prerequisite for the retainment of skyrmions. As shown in Supplementary Fig. 14c, a considerable amount of skyrmions are still present even when strain is set as zero and D remains to be 0.585 mJ/m^2.

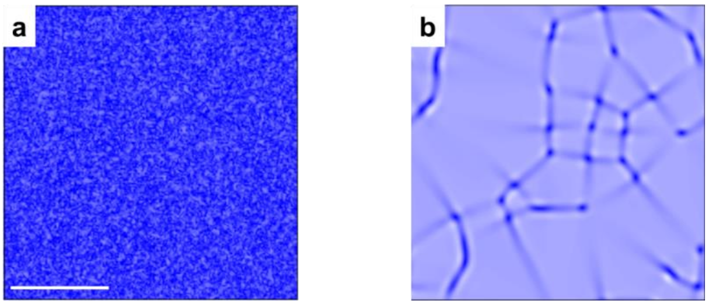

Supplementary Fig. 15 | Magnetization switching in single-crystalline model. Magnetization distribution at **a**, initial state without strain, and **b**, equilibrium state after applying strain $\varepsilon_{[1-10]} = \varepsilon_{[110]} = -0.189\%$. The scale bar is 1 μm.

Remarkably, we show that if removing all the spatial variance of the magnetic parameters including the axis of uniaxial magnetocrystalline anisotropy, magnetocrystalline anisotropy constant and the interfacial DMI strength from the model set up (see Methods section), which is in effect describing a single-crystalline magnetic system, the initially nearly [001] magnetization in Supplementary Fig. 15a will be switched to film plane with an average $\langle m_z \rangle = 0.54$ upon applying the same amount of strains in Fig. 3f in the Main Text, where some antivortex cores instead of skyrmions can be found (Supplementary Fig. 15b). Therefore, we introduce the polycrystalline model with a finite grain size to simulate the skyrmion creation process.

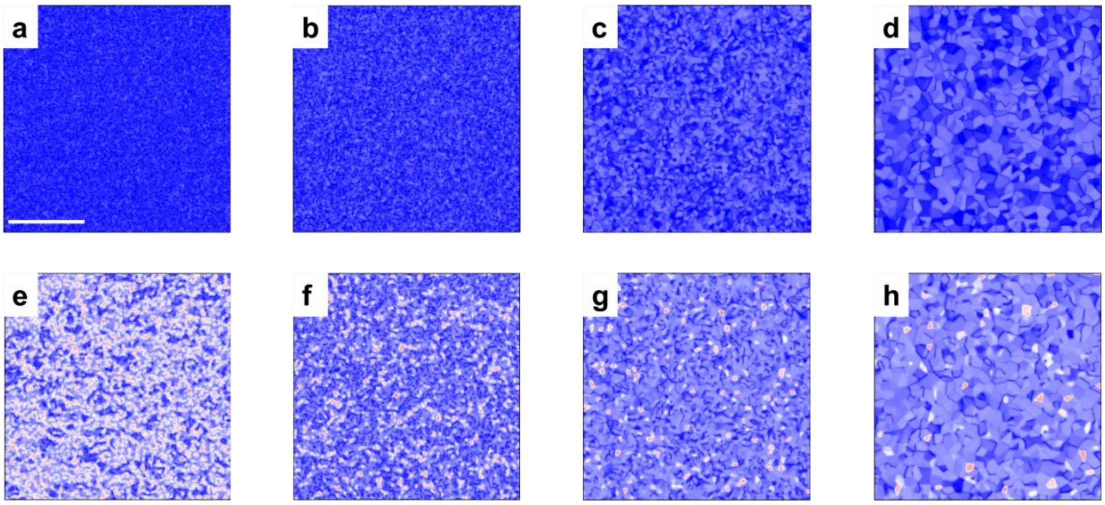

Supplementary Fig. 16 | Effect of grain size. a-d, initial state of magnetization distribution relaxed from a uniform distribution along [001] under zero strains, with grain size of 10 nm, 20 nm 50 nm and 100 nm. **e-h**, Skyrmions appear after applying strain $\varepsilon_{[1-10]} = \varepsilon_{[110]} = -0.189\%$, with grain size of 10 nm, 20 nm 50 nm and 100 nm. The model set up is identical to that described in Methods section of the Main Text expect the grain size. The scale bar is 1 μm.

Supplementary Fig. 16 shows the skyrmions creation in the polycrystalline magnetic layer with different grain sizes, as can be seen, the use of larger grain size yields larger but fewer skyrmions. For the results shown in Figure 3, a mean grain size of 20 nm is used, which is also used in other work.[4]

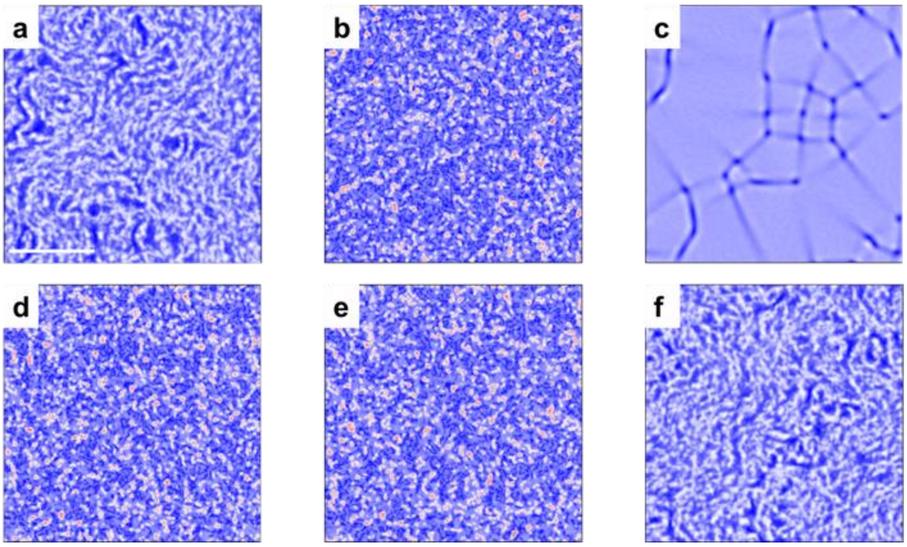

Supplementary Fig. 17 | Necessity and sufficiency of spatially varied parameters for skyrmion creation. The magnetization distributions after applying strain $\varepsilon_{[1-10]} = \varepsilon_{[110]} = -0.189\%$, starting from same initial state, with **a**, only K_U spatially varied; **b**, only the axis of uniaxial magnetocrystalline anisotropy spatially varied; **c**, only D spatially varied; **d**, both K_U and the axis of uniaxial magnetocrystalline anisotropy spatially varied; **e**, both D and the axis of uniaxial magnetocrystalline anisotropy spatially varied; **f**, both K_U and D spatially varied as described in the Method. The scale bar is 1 μm.

In Supplementary Fig. 17, we performed six independent groups of simulations for the skyrmion creation. In Supplementary Fig. 17a-c, the three parameters (K_U, the axis of uniaxial magnetocrystalline anisotropy, and D) are varied independently (when one parameter is varied, the other two are set to be spatially uniform), and in Supplementary Fig. 17d-f, two of the parameters (K_U, the axis of uniaxial magnetocrystalline anisotropy, and D) are varied simultaneously, the other one is set to be spatially uniform. It is found that the spatial variation in the axis of uniaxial magnetocrystalline anisotropy is both necessary and sufficient for strain-mediated

skyrmion creation.

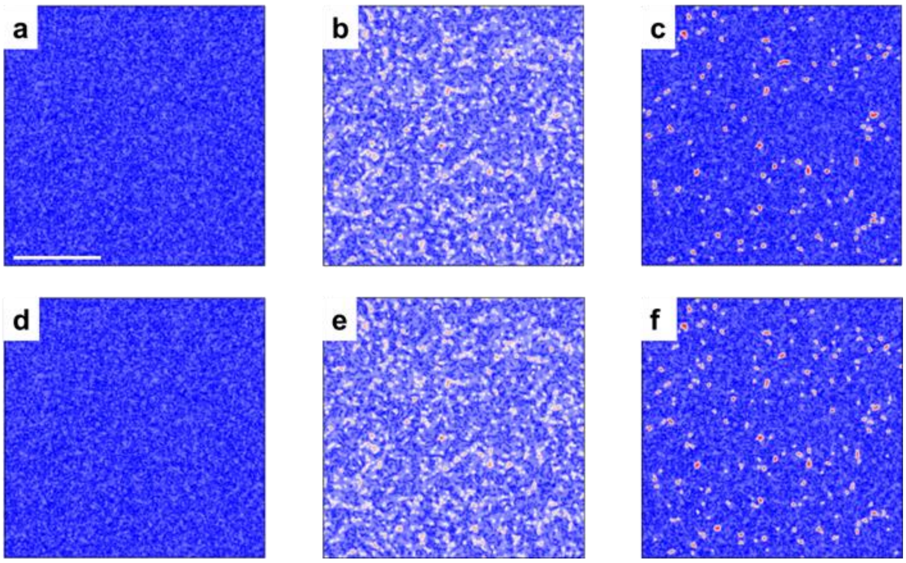

Supplementary Fig. 18 | Scaling test for computational cell size. Magnetization distribution at initial state with E = +0 kV/cm, after applying strain $\varepsilon_{[1-10]} = \varepsilon_{[110]} = -0.189\%$ with E = -4 kV/cm and after reducing the strain to $\varepsilon_{[1-10]} = \varepsilon_{[110]} = -0.034\%$ with E = -0 kV/cm, in a 3 μm×3 μm simulation system, **a-c**, with cell size of 3 nm, and **d-f**, with cell size of 1.5 nm. The set-up is identical to that described in Method section. The scale bar is 1 μm.

A scaling test was performed to ensure the size of the discretized computational cells is small enough, as shown in Supplementary Fig. 18. For the simulation results shown in the first row, $\Delta x = \Delta y = 3$ nm and $n_x = n_y = 1000$ are used, and for simulation results shown in the second line, $\Delta x = \Delta y = 1.5$ nm and $n_x = n_y = 2000$ are used. As can be seen in Supplementary Fig. 18, the simulations with different cell size yield very similar spatial distribution of the magnetization. Considering longer computation time for more discretized computational cells, we set $\Delta x = \Delta y = 3$ nm and $n_x = n_y = 1000$ in

the simulations for Fig. 3.

In addition, when simulating strain-mediated deformation and annihilation of one single skyrmion (Fig. 4-5), we adopt the single-crystalline model with $\Delta x = \Delta y = 0.5$ nm for higher precision and $n_x = n_y = 600$ for faster computation.

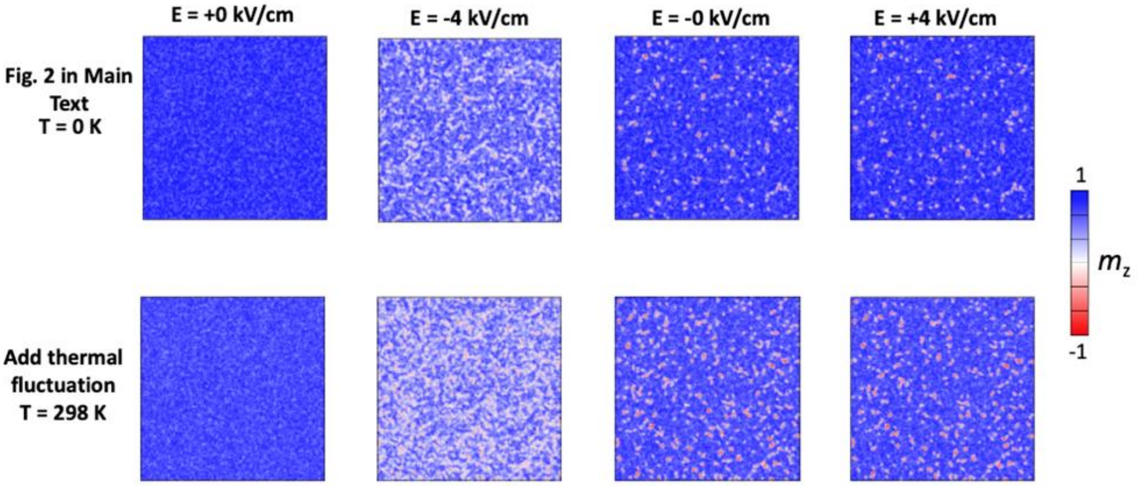

Supplementary Fig. 19 | Micromagnetic simulation results with/without thermal fluctuation.

In the main paper, we set T = 0 K (that is, excluding the thermal fluctuation) for performing a clean free energy analysis on the strain-mediated skyrmion creation. We checked the influence of $\mathbf{B}_{\text{therm}}$ (T = 298 K) on the strain-mediated skyrmion creation process. The Supplementary Fig. 19 compares the results under T = 0 K (the first row, corresponding to Fig. 3e-h in the main paper) and T = 298 K, respectively. All other settings are kept the same. As can be seen, the overall behavior of the strain-mediated creation of skyrmions under T = 298 K is similar to both the simulation results obtained under T = 0 K, hence being similar to experimental observations (see Fig. 3a-d in the main paper). Regarding the details of the switching process, two observations are noted

below. First, the addition of $\mathbf{B}_{\text{therm}}$ (T=298 K) introduces white noise into the magnetization distribution. Second, compared to the case $\mathbf{B}_{\text{therm}} = 0$ (T = 0 K), there are more skyrmions created when in-plane compressive strain is applied with E = -4 kV/cm, and more skyrmions retained when the electric field is turned off (E = -0 kV/cm).

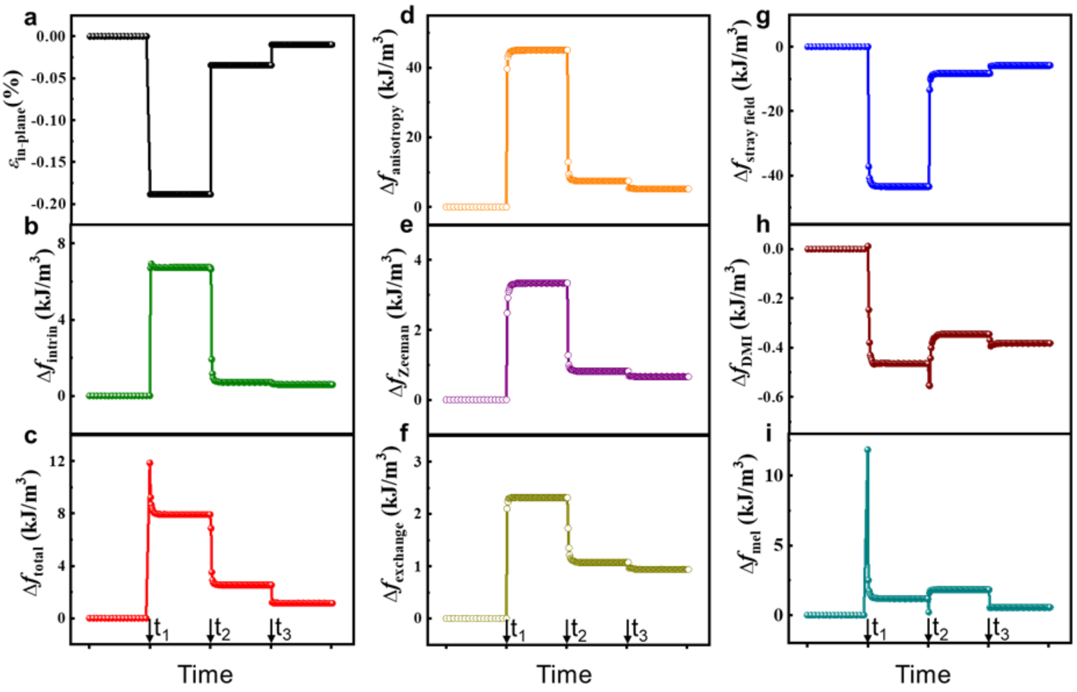

Supplementary Fig. 20 | Evolution of the applied in-plane biaxial compressive strain and the energy densities in the process from Fig. 3e to Fig. 3h. The evolution of **a**, in-plane biaxial compressive strain, **b-i**, change of the intrinsic free energy density, total free energy density, anisotropy energy density, Zeeman energy density, exchange energy density, stray field energy density, magnetoelastic energy density and DMI energy density. The values of energies before strain is applied at $t = t_1$ are taken as reference zero point.

As shown in Supplementary Fig. 20, when the strain $\varepsilon_{[1-10]} = \varepsilon_{[110]} = -0.189\%$ is

applied at $t = t_1$ with electric field E = -4 kV/cm, the magnetoelastic energy density Δf_{mel} and total energy density Δf_{tot} increase steeply by about 12 kJ/m^3. Then, the decrease in Δf_{mel} and increase in $\Delta f_{intrin} = \Delta f_{tot} - \Delta f_{mel}$ follow immediately after the process of skyrmion creation starts, in this process, the total energy density decreases by about 4 kJ/m^3 because the magnitude of the decrease in Δf_{mel} is larger than the increase in Δf_{intrin}, therefore, the decrease in Δf_{mel} is a driving force of the skyrmions creation. More specifically, among all contributions to Δf_{intrin}, DMI energy density Δf_{DMI} and magnetostatic stray field energy density $\Delta f_{stray\ field}$ also decrease, which drive the creation of skyrmions along with the release of the Δf_{mel}.

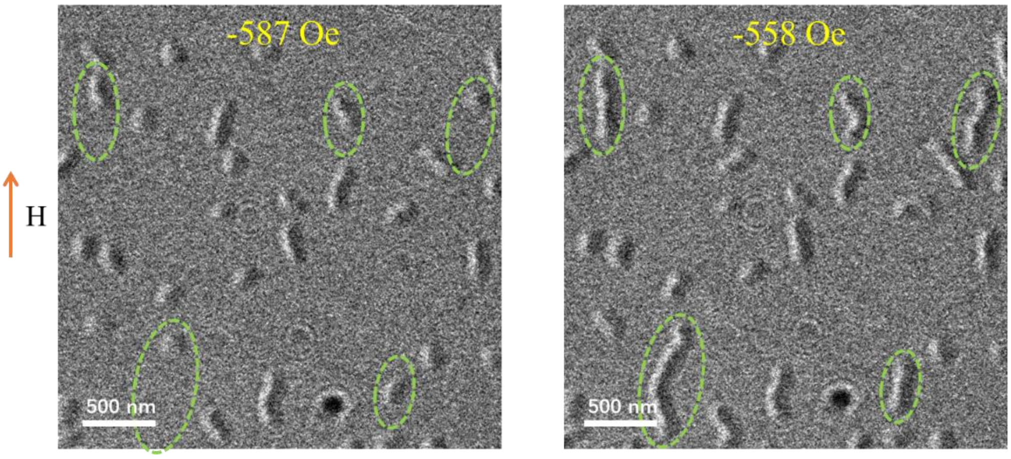

Supplementary Fig. 21 | In-situ L-TEM observation. The images were taken at a tilt angle of α=20.06° and β=20.39°, and the arrow indicates the in-plane magnetic field direction.

As demonstrated by Senfu Zhang et al, observeaton of the asymmetric domain expansion under an in-plane magnetic field by L-TEM can be used to get the chirality of Néel-type skyrmions[4]. Therefore, we observed asymmetric domain expansion under an in-plane magnetic field by L-TEM. For Pt/Co/Ta multilayers, Senfu Zhang et al

showed that "on decreasing the magnetic field, individual skyrmions appear to subsequently evolve into snake-like structures growing in the direction opposite to the in-plane magnetic field", which illustrate that these skyrmions have left-handed chirality[4]. As shown in Supplementary Fig. 21, we indicated the changes in the images with the green dashed ellipses. The directions that the snake-like structures preferred to grow along are also opposite to that of the in-plane field in our work, consistent with that of Senfu Zhang et al's wrok[4]. This proves that the skyrmions in our Pt/Co/Ta multilayers actually have left-handed chirality, which has also been demonstrated by the previous reports[9, 10].

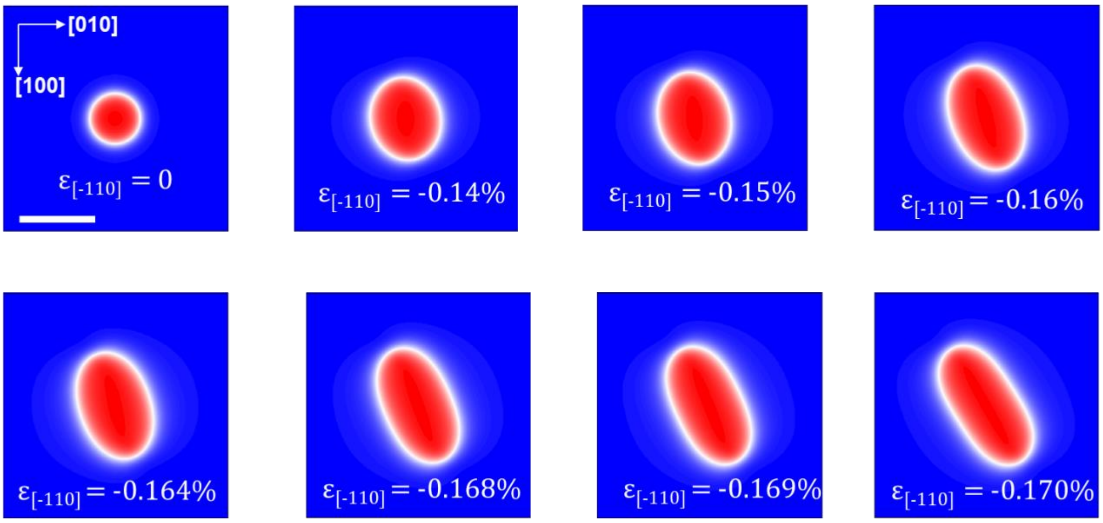

Supplementary Fig. 22 | A series of skyrmion morphology under different in-plane uniaxial compressive strain. The scale bar is 100 nm.

Although we use the in-plane isotropic strain assumption in Fig. 2, the local strain can be anisotropic due to the different FE domain switching[5,6]. The simulation result shows skyrmion deformation caused by the in-plane uniaxial compressive strain.

References

1. Liu, H. et al. Critical role of monoclinic polarization rotation in high-performance perovskite piezoelectric materials. *Phys. Rev. Lett.* **119,** 017601 (2017).
2. Fu, H. & Cohen, R. E. Polarization rotation mechanism for ultrahigh electromechanical response in single-crystal piezoelectrics. *Nature* **403,** 281-283 (2000).
3. Chen, Q. et al. Electric-field control of phase separation and memory effect in $Pr_{0.6}Ca_{0.4}MnO_3$/Pb $(Mg_{1/3}Nb_{2/3})_{0.7}Ti_{0.3}O_3$ heterostructures. *Appl. Phys. Lett.* **98,** 172507 (2011).
4. Zhang, S., Zhang, J., Wen, Y., Chudnovsky, E. M. & Zhang, X. Determination of chirality and density control of Néel-type skyrmions with in-plane magnetic field. *Commun. Phys.* **1,** 36 (2018).
5. Ba, Y. et al. Spatially resolved electric-field manipulation of magnetism for CoFeB mesoscopic discs on ferroelectrics. *Adv. Funct. Mater.* **28,** 1706448 (2018).
6. Ghidini, M. et al. Shear-strain-mediated magnetoelectric effects revealed by imaging. *Nat. Mater.* **18,** 840-845 (2019).
7. Ma, J. et al. Controllable conductive readout in self-assembled, topologically confined ferroelectric domain walls. *Nat. Nanotechnol.* **13,** 947-952 (2018).
8. Gallardo, R. A. et al. Reconfigurable Spin-Wave Nonreciprocity Induced by Dipolar Interaction in a Coupled Ferromagnetic Bilayer. *Phys. Rev. Appl.* **12,** 034012 (2019).
9. Saha, S. et al. Formation of Néel-type skyrmions in an antidot lattice with

perpendicular magnetic anisotropy. *Phys. Rev. B* **100,** 144435 (2019).

10. Woo, S. et al. Observation of room-temperature magnetic skyrmions and their current-driven dynamics in ultrathin metallic ferromagnets. *Nat. Mater.* **15**, 501–506 (2016).
11. Grungberg, P. et al. Magnetostatic spin-wave modes of a heterogeneous ferromagnetic double layer. *J. Appl. Phys.* **52**, 6824 (1981).
